# Isolated Facial Diplegia: A Rare Presentation of Guillain-Barré Syndrome

**DOI:** 10.7759/cureus.51126

**Published:** 2023-12-26

**Authors:** Taoufik Boubga, Abdellah Taous, Tarik Boulahri, Maha Ait Berri

**Affiliations:** 1 Department of Neurology, Military Hospital Moulay Ismail of Meknès, Meknès, MAR; 2 Department of Neurology, Faculty of Medicine and Pharmacy of Fez, Fez, MAR; 3 Department of Neurology, Sidi Mohamed Ben Abdellah University, Fez, MAR

**Keywords:** bell's palsy, neurophysiological studies, anti-ganglioside antibodies, guillain-barré syndrome, facial paralysis

## Abstract

Guillain-Barré syndrome is an autoimmune condition typically characterized by progressive areflexic ascending motor deficit and paresthesia. However, atypical presentations, such as isolated facial diplegia, are rare and diagnostically challenging.

We describe a unique case of Guillain-Barré syndrome in a 46-year-old male patient, presenting as isolated bilateral facial paralysis without preceding medical history. Symptoms included tongue heaviness, loss of taste, dysarthria, and inability to close eyelids. A neurological examination confirmed bilateral facial paralysis. Laboratory tests and cerebrospinal fluid analyses were unremarkable, except for albumin-cytological dissociation. Electromyography revealed severe demyelinating damage to facial nerves. The patient responded well to intravenous immunoglobulin therapy.

This case highlights the necessity of considering Guillain-Barré syndrome in patients with isolated facial diplegia. A thorough clinical evaluation, supported by laboratory and electromyographic findings, is crucial for accurate diagnosis and effective treatment. Early identification and intervention are key for optimal outcomes in these atypical presentations.

## Introduction

Guillain-Barré syndrome represents an autoimmune pathology that impacts the peripheral nervous system, specifically affecting the peripheral nerves and their root structures. This condition is commonly characterized by a rapidly progressing, areflexic flaccid ascending motor deficit, which typically starts in the lower extremities and can ascend upwards. Despite this usual presentation, Guillain-Barré syndrome can exhibit atypical clinical manifestations. The significance of recognizing these unusual presentations is particularly emphasized by cases such as the one observed in our patient, who exhibited a rare form of the disease. In this instance, the patient presented with isolated bilateral facial paralysis, an uncommon symptom in the spectrum of Guillain-Barré syndrome manifestations. This highlights the diverse clinical spectrum of the syndrome and the need for heightened awareness and diagnostic acumen to identify and manage such atypical cases effectively.

## Case presentation

We report the case of a 46-year-old male patient with no significant medical history or recent episodes of infection. He received vaccinations as per the national guidelines in effect. The patient sought medical consultation due to experiencing a sensation of heaviness in his tongue, which started two days before the consultation, accompanied by a loss of taste. He also reported dysarthria and an inability to completely close his eyelids, without any other symptoms, including paresthesia.

**Figure 1:**
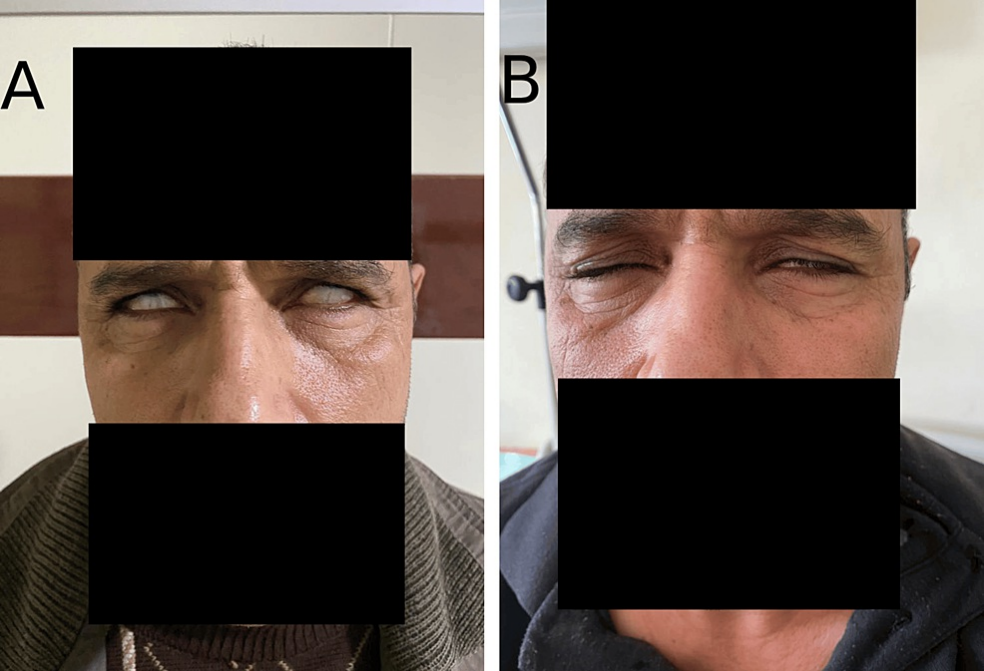
Photograph of the patient admitted for facial diplegia with Guillain-Barré syndrome. A: Patient at admission with bilateral peripheral facial paralysis. B: Resolution of the symptomatology one month after presentation.

Upon conducting a neurological examination, the patient was found to have isolated bilateral peripheral facial paralysis (Figure 1A). This finding was significant as the other cranial nerves appeared to be functioning normally. The patient did not show any signs of muscle weakness or sensory disturbances, and there was no evidence of dysphagia or respiratory muscle weakness. His osteotendinous reflexes were observed to be normal in all four limbs. Furthermore, there were no signs of ataxia or any gait disturbances. The remaining systemic examination was unremarkable.

Extensive laboratory testing was undertaken, including routine blood tests and specific serological tests for diseases such as HIV, Lyme disease, hepatitis, and syphilis. All of these tests returned negative results. An examination of the patient’s cerebrospinal fluid was also performed, which revealed an albuminocytological dissociation but did not indicate any infectious processes.

Further immunological testing was conducted, focusing on the presence of any abnormal antibodies, including anti-ganglioside IgG and IgM types. However, these tests did not reveal any anomalies.

Imaging studies were also performed. A brain MRI was conducted, which did not reveal any significant abnormalities.

Electromyography and nerve conduction velocity tests were conducted three days after the onset of the symptoms.

The motor nerve conduction study included the median, ulnar, common peroneal, and posterior tibial nerves. The results were within normal ranges, showing latencies approximately between 2.5 and 4 ms and conduction velocities from 45 to 60 m/s across both sides. This suggests healthy nerve function.

In the sensory nerve conduction study, the median, ulnar, and sural nerves also demonstrated normal functioning. The latencies ranged from 1.5 to 3 ms, and the velocities were between 45 and 65 m/s. These results indicate consistent and healthy nerve responses on both sides.

However, the facial nerve study revealed signs of demyelination on both sides. The right facial nerve (r. auricularis) exhibited a latency of 4 ms and an amplitude of 4.5 mV, while the left had a latency of 3.6 ms with an amplitude of 4.5 mV.

Lastly, the blink reflex study showed an abnormal bilateral response, which showed right‐sided absent ipsilateral R1 and R2, with absent contralateral R2, and absent ipsilateral and contralateral responses on the left side.

The diagnosis of Guillain-Barré syndrome was established in this patient based on the electromyography profile showing demyelination in the facial nerves and the presence of albuminocytological dissociation in the cerebrospinal fluid, coupled with the absence of abnormalities in other examinations.

The patient, who weighed 74 kg, received intravenous immunoglobulin (IVIG) therapy, dosed at 0.4 g/kg daily for five days, amounting to approximately 30 grams per day. The IVIG infusions were closely monitored for any adverse reactions. Complementing the IVIG therapy, ocular lubricants and eye patches were used to manage the specific symptoms of facial diplegia. Regular follow-up appointments were essential to assess the patient's progress and response to the treatment.

This treatment led to a significant improvement in his condition (Figure 1B). This improvement was both clinically observable and corroborated by follow-up neurological assessments.

## Discussion

Unilateral facial nerve paralysis is a relatively common condition, with about half of the cases being of unknown origin, termed idiopathic. In contrast, bilateral facial nerve paralysis is notably rarer, occurring at a rate of one in 500,000 individuals. Intriguingly, only 20% of these cases are idiopathic [1], while the remainder are associated with a range of potentially serious medical conditions.

Among the common causes of bilateral facial nerve paralysis are Lyme disease, Guillain-Barré syndrome, sarcoidosis, diabetes mellitus, acute leukemia, porphyria, HIV infection, and multiple sclerosis [2]. These conditions highlight the diverse etiological factors that can contribute to this neurological manifestation.

In the diagnostic process, a rigorous clinical interview combined with a comprehensive neurological examination is essential. This approach is crucial in establishing a differential diagnosis that distinguishes between the various possible causes of facial nerve paralysis. In the context of the research study being discussed, two key diagnostic indicators are highlighted for Guillain-Barré syndrome: the presence of albuminocytological dissociation, which is observed in about 90% of Guillain-Barré syndrome cases [3], and the findings from electromyography, which plays a pivotal role in confirming the diagnosis. However, it is important to note that electromyography can sometimes yield non-specific results, which may complicate the diagnostic process [4].

In cases of bilateral facial paralysis, a notable observation is that they are generally not linked to the presence of anti-ganglioside antibodies, as was observed in our patient. However, there is an association with anti-GM2 antibodies, and more rarely, with anti-GD1a antibodies. The latter are typically linked to axonal forms of neurological disorders [5].

The management of Guillain-Barré syndrome involves both symptomatic care and specific etiopathogenic treatments. The mainstays of such treatments include the administration of IVIGs or the undertaking of plasmapheresis. Although both treatment modalities have been found to have similar effectiveness, combining these treatments does not yield additional benefits and is not recommended [6].

## Conclusions

In summary, this case of Guillain-Barré syndrome, presenting as isolated facial diplegia, illustrates the complexity and variability of the syndrome. It underscores the necessity of a thorough diagnostic approach, combining clinical examination with specific tests, for effective identification of atypical Guillain-Barré manifestations. This case highlights the potential for positive outcomes with prompt and appropriate therapy, even in rare presentations of the condition.
